# Eyes-state-dependent alterations of magnetoencephalographic connectivity associated with delayed recall in Alzheimer’s disease via graph theory approach

**DOI:** 10.3389/fpsyt.2023.1272120

**Published:** 2023-10-24

**Authors:** Keigo Yuasa, Tetsu Hirosawa, Daiki Soma, Naoki Furutani, Masafumi Kameya, Masuhiko Sano, Koji Kitamura, Minehisa Ueda, Mitsuru Kikuchi

**Affiliations:** ^1^Department of Psychiatry and Neurobiology, Graduate School of Medical Science, Kanazawa University, Kanazawa, Japan; ^2^Research Center for Child Mental Development, Kanazawa University, Kanazawa, Japan

**Keywords:** Alzheimer’s disease, magnetoencephalography, graph theory, delayed recall, eyes-open, eyes-closed

## Abstract

**Introduction:**

Alzheimer’s disease (AD) is a neurodegenerative disorder characterized by memory impairment and cognitive decline. Electroencephalography (EEG) and magnetoencephalography (MEG) studies using graph theory show altered “Small-Worldness (SW)” properties in AD. This study aimed to investigate whether eye-state-dependent alterations in SW differ between patients with AD and healthy controls, considering the symptoms of AD.

**Methods:**

Nineteen patients with AD and 24 healthy controls underwent MEG under different conditions (eyes-open [EO] and eyes-closed [EC]) and the Wechsler Memory Scale-Revised (WMS-R) with delayed recall. After the signal sources were mapped onto the Desikan–Killiany brain atlas, the statistical connectivity of five frequency bands (delta, theta, alpha, beta, and gamma) was calculated using the phase lag index (PLI), and binary graphs for each frequency band were constructed based on the PLI. Next, we measured SW as a graph metric and evaluated three points: the impact of AD and experimental conditions on SW, the association between SW and delayed recall, and changes in SW across experimental conditions correlated with delayed recall.

**Results:**

SW in the gamma band was significantly lower in patients with AD (*z* = −2.16, *p* = 0.031), but the experimental conditions did not exhibit a significant effect in any frequency band. Next, in the AD group, higher scores on delayed recall correlated with diminished SW across delta, alpha, and beta bands in the EO condition. Finally, delayed recall scores significantly predicted relative differences in the SW group in the alpha band (*t* = −2.98, *p* = 0.009).

**Discussion:**

Given that network studies could corroborate the results of previous power spectrum studies, our findings contribute to a multifaceted understanding of functional brain networks in AD, emphasizing that the SW properties of these networks change according to disease status, cognitive function, and experimental conditions.

## 1. Introduction

Alzheimer’s disease (AD), the leading cause of dementia, is a neurodegenerative disorder typically occurring mid-life (1). It is characterized by an escalating progression of memory impairment and cognitive decline (1). Globally, an estimated 46 million individuals are diagnosed with AD, predicted to escalate to 13 million in the United States and 130 million worldwide by 2050, owing to the expanding elderly population (2, 3). The fundamental neuropathological changes characteristic of AD involve the extracellular deposition of amyloid beta peptide and the intracellular formation of neurofibrillary tangles constituted by hyperphosphorylated tau protein and the development of neuritic plaques (1, 2). In the early stages of AD, amyloid-beta instigates alterations in both GABAergic (4) and glutamatergic functions (5–9) in a manner that is dependent on tau protein (3, 10). Synaptic dysfunction is widely acknowledged as the primary anatomical correlate of initial cognitive impairment in AD (11, 12) and is detectable before the onset of noticeable cognitive decline and neuronal cell death (13). These alterations disrupt effective neuronal communication, culminating in progressive and extensive neural and synaptic loss, predominantly in the cerebral cortex and hippocampus (14, 15).

Among the methodologies currently used to explore human brain function, electroencephalography (EEG) and magnetoencephalography (MEG) offer a direct, real-time reflection of brain electrical activity at the synaptic level (16). As expected from AD pathology, specifically synaptic dysfunction and characteristic alterations in resting-state brain oscillations (rhythms) have been observed in patients with the disease. Widespread consensus exists on the typical spectral changes seen in AD. These changes involve reduced fast oscillations (alpha, beta, and gamma frequencies) over posterior regions and a general increase in slow rhythms (delta and theta frequencies). These are among the most frequently reported findings from EEG/MEG studies on resting-state activity in AD (17–22). The reactivity of alpha waves or their suppression in response to eye-opening also exhibits progressive impairment from normal aging to mild AD, a decline that aligns with cognitive deterioration (23).

An alternative approach examines functional disconnections among neurons in various brain regions; this was performed by investigating the correlations between brain activity signals recorded from these disparate areas. The foundation of this method rests on the belief that these correlations, at least to some extent, reflect functional interactions occurring among different brain areas. This concept of functional connectivity (24) represents the statistical interdependencies between brain activity signals and is often used as a provisional measure of functional interactions. Broadly, findings from functional connectivity analyses suggest a trend of reduced connectivity across many frequency bands and pairs of brain regions in patients with AD. Babiloni et al. documented reduced connectivity in the alpha band pertaining to both posterior interhemispheric and widespread intrahemispheric connections (25). Hata et al. reported lower connectivity in the delta band for most cortical region pairs (26). These insights were derived from a string of connectivity comparisons between diverse pairs of brain regions. However, these connectivity abnormalities’ topological patterns or distributions may be equally significant. A more holistic understanding of its wide-ranging impact on interlinked brain regions can be achieved by probing the implications of AD on the overarching neural networks.

In the context of EEG and MEG studies, the brain is envisioned as a network of discrete regions that interact dynamically over time. The properties of this complex network can be elucidated by applying graph theory (27). This framework simplifies the brain into a “graph”— a construct where the nodes represent unique brain regions, and the edges symbolize the connectivity between these regions (28). Graph theory introduces measures that encapsulate the attributes of graphs as singular numerical values, thereby offering a more manageable way to understand the complex characteristics of these networks. Notably, the mean clustering coefficient (C) and the average shortest path length (L) are recognized for their simplicity, reliability, and widespread use. The mean clustering coefficient (C) gauges the degree to which connected nodes form local clusters, with a higher C indicating a greater propensity for the brain to process information locally, an activity known as functional segregation (29).

Conversely, the average shortest path length (L) represents the mean number of edges that must cross from one node to another, with the average taken over all the potential node pairs. Thus, a shorter L signified the tendency of the network to integrate information from distant brain regions, a process referred to as functional integration (29). Structural (30, 31) and functional (32, 33) studies suggest that healthy human brain networks typically exhibit high C and short L values. This unique combination of properties facilitates efficient information processing within the intricate brain network, indicating an optimal balance between functional integration and segregation (34). These networks, characterized as “small-world” networks (34), represent an elegant equilibrium between local specialization and global integration, making them a robust model for elucidating the structural intricacies of the human brain. The characteristic known small-worldness (SW) quantifies the degree to which a network displays small-world properties, which are defined as the ratio of normalized C to normalized L. The concept of SW holds particular intrigue, as the healthy human brain is well-established as functioning within the framework of a small-world network (30–33).

Numerous studies have harnessed the power of graph theory in combination with EEG to compare the functional brain networks of healthy aging individuals with those of patients with AD. However, it is crucial to interpret the results of these studies with caution because of differences in participant characteristics, the selection of connectivity measures (e.g., lagged linear coherence or synchronization likelihood), and the types of graphs used (e.g., binary vs. weighted). Despite these limitations, a consensus seems to hold that among various graph theoretical measures, the functional brain network of patients with AD exhibits altered SW compared to healthy controls (35–43). Specifically, when these networks are constructed from resting-state recordings under eyes-closed [EC] conditions, patients with AD tend to exhibit reduced SW in the delta and theta frequency bands (35–37, 41, 42), along with the beta band (36, 40, 41), relative to healthy individuals. In contrast, patients with AD showed elevated SW within the alpha band (36, 41, 42). Strengthening these observations, patients with mild amnestic cognitive impairment, particularly those who later progressed to AD, demonstrated analogous patterns of SW alterations. They display reduced SW in the delta, beta, and gamma bands and elevated SW in the alpha band compared to individuals who do not transition to AD (38). The relationship between altered SW and AD symptoms, like memory impairment, could be hypothesized. However, it is crucial to emphasize that only a few studies have investigated the correlation between altered SW and AD symptoms. The study by Vecchio et al. (42) served as a notable exception, identifying a positive correlation between SW in the gamma band and working memory as assessed by the digit span task. Unfortunately, this study did not examine the relationship between SW and other memory function dimensions. For instance, delayed recall is one of the most potent neuropsychological indicators of transition from MCI to AD (44). Therefore, research on this facet may provide invaluable insights into the early detection and progression of AD.

Given the progressive impairment in the reactivity of alpha waves, or more specifically, their suppression in response to eye-opening, observed in patients with AD (23), an exploration of the variances in the SW properties of functional brain networks between EC and eyes-open (EO) states in patients with AD might be insightful. However, this issue has only been addressed by Miraglia et al. (35). Miraglia et al. reported analogous patterns of SW changes between the EC and EO states in patients with AD and healthy controls. Specifically, both groups demonstrated reduced SW in the EC state compared to that in the EO state within the higher alpha band (10.5–13 Hz) while showing increased SW for the EC state compared to the EO state in the high-beta (20–30 Hz) and gamma bands (30–45 Hz). Intriguingly, healthy controls also exhibited decreased SW in the EC state compared to that in the EO state within the lower alpha band (8–10.5 Hz); however, this reactivity was absent in the AD group. Regrettably, Miraglia et al. (35) did not examine the interaction effect between disease condition (AD vs. healthy controls) and state (i.e., EC vs. EO) on SW. Whether state-dependent alterations in SW differ between patients with AD and healthy controls remains unclear. Furthermore, they did not delve into the relationship between changes in SW and the symptoms of AD, an exploration that could be informative.

This study pursued three main objectives to supplement existing evidence. First, we aimed to evaluate the impact of AD (relative to healthy controls) and different experimental conditions (EO vs. EC) on SW while accounting for potential age effects (45). Second, we sought to determine whether SW in the functional brain network was associated with delayed recall after adjusting for age-related effects. Finally, we investigated whether changes in SW across the experimental conditions correlated with delayed recall, controlling for age. We hypothesized that patients with AD exhibit different trends in SW changes between experimental conditions compared to healthy controls and that disease specificity can be predicted from the changes in experimental conditions and neuropsychological tests. For this investigation, we opted to employ binary graph methods because their results tend to be more straightforward to interpret (46).

This study pursued three main objectives to supplement existing evidence and contribute to the growing body of knowledge. First, we aimed to evaluate the impact of AD (relative to healthy controls) and different experimental conditions (EO vs. EC) on SW while accounting for potential age effects (45). Second, we sought to determine whether SW in the functional brain network was associated with delayed recall after adjusting for age-related effects. Finally, we investigated whether changes in SW across the experimental conditions correlated with delayed recall, controlling for age.

We explicitly acknowledge the exploratory nature of this study, and therefore, our hypotheses were formulated on a provisional basis. Firstly, we hypothesized that patients with AD may exhibit discernible variations in SW compared to healthy controls, with SW fluctuating between the EO and EC conditions. Secondly, we anticipate the presence of a relationship between the SW attributes of the brain network and delayed recall, encompassing both patients diagnosed with AD and those without. Lastly, we proposed that a correlation could potentially be observed between the extent of SW variations across EO and EC conditions and the delayed recall outcomes in both patient groups.

To facilitate a clear and straightforward interpretation of our preliminary findings, we have chosen to employ binary graph methods in this investigation (46). This methodology hold the promise not only of revealing deeper insights but also of laying the groundwork for subsequent, more conclusive research in the intricate domain of neurological network analysis.

## 2. Methods

### 2.1. Experimental design

The present observational study included a control group. We enrolled 43 participants: 19 adults diagnosed with AD and 24 age-matched healthy controls. All participants underwent two MEG recording sessions under different conditions: open and closed eyes. The sequence of these conditions was randomized across participants to control for potential order effects.

Our analysis focused on comparing the functional brain networks of AD and control groups under both conditions. Specifically, we investigated the differences between these groups in the EO and EC states. Additionally, we examined the changes in the functional brain networks between these two conditions for each group.

### 2.2. Participants

Individuals with AD were recruited from the Kanazawa University Hospital and its associated hospitals. They fulfilled the diagnostic criteria for probable AD proposed by the National Institute of Neurological and Communicative Diseases and Stroke/AD and Related Disorders Association workgroup (47). All participants were screened using neurological, serological, and magnetic resonance imaging (MRI) studies to exclude other medical conditions that could cause dementia. The duration of AD, defined as the time from the onset of symptoms, was obtained through interviews with family members. Twelve patients were administered a daily dose of donepezil hydrochloride (5 mg). Apart from donepezil hydrochloride, none of the patients were taking any other medications that affected the central nervous system, including antipsychotics, anticholinergics, antidepressants, anticonvulsants, benzodiazepines, cerebral metabolic activators, or cerebral vasodilators. A team of experienced psychiatrists and psychologists evaluated the patients’ memory abilities using the Wechsler Memory Scale-Revised (WMS-R) (48), while AD progression was assessed using the Functional Assessment Staging Test (49) and Clinical Dementia Rating (50).

The 24 healthy controls had no personal or family history of psychiatric or neurological disorders. All the individuals in the control group were functionally independent. They underwent the Mini-Mental State Examination (51) and the WMS-R to confirm that they maintained normal memory function. None of their WMS-R sub-scores fell below the 1.5 standard deviations from the normal range. We adapted the 1.5 standard deviation threshold in accordance with the criteria established by Petersen and Morris (52). These criteria operationally define objective cognitive impairment across various subtypes of MCI. As per these criteria, individuals were categorized as “normal” if no neuropsychological measure exhibited a deviation of more than 1.5 SD below the age-appropriate norms in any cognitive domain.

All participants agreed to participate in the study and were fully informed of its nature and objectives. Written informed consent was obtained from all participants before their enrollment in the study. In the case of individuals within the AD group, the consent process involved a dual approach: initial consent was solicited from the individuals themselves, who, despite having mild dementia, exhibited sufficient decision-making capacity, as determined through clinical interviews. Concurrently, consent was also obtained from their family members or legal guardians, further ensuring the ethical integrity of the research process. The Ethics Committee of the Kanazawa University Hospital approved this study.

The participants in the current study partially overlapped with those in our previous research (53); however, none of the results presented in this study overlapped with the findings of the previous research. Moreover, the focus and objectives of the previous study were distinct from those of the current study.

### 2.3. Assessment of delayed recall: WMS-R

The WMS-R is an extensively used neuropsychological instrument designed to assess the various dimensions of memory function in adults (48). Developed by David Wechsler, it enhanced its predecessor, the original WMS, by providing a comprehensive and differentiated evaluation of memory, thereby augmenting our understanding of individuals’ memory capacity and potential deficits.

The WMS-R comprises several subtests designed to measure diverse facets of memory, including verbal and visual memory, attention/concentration, and delayed recall. Each subtest generates individual scores that can be analyzed separately. In this study, we focused primarily on delayed recall subtests. A higher delayed recall score signifies superior overall performance in recalling information after a delay (48).

We concentrated on delayed recall because of its marked significance in AD. The scores on this subtest typically decline significantly in patients with AD. Furthermore, measures of delayed recall have been identified as the most robust neuropsychological predictors of conversion from mild cognitive impairment to Alzheimer’s (44). Studying this aspect can yield valuable insights into this disease’s early detection and progression.

### 2.4. MEG and MRI recordings

MEG and MRI recording protocols followed those established in our previous study (53). All the participants underwent two MEG recording sessions, each lasting 2 min, under different conditions: EO and EC.

MEG recordings were performed using a 160-channel whole-head coaxial gradiometer system (PQ 1160C; Yokogawa Electric Corp., Kanazawa, Japan) inside a magnetically shielded room at the MEG Center of Yokogawa Electric Corp. Each gradiometer was equipped with 15.5-mm-diameter pick-up coils set apart at a distance of 25 mm. Data sampling was performed at a rate of 5,000 Hz per channel with a band-pass filter ranging from 0.1–3,000 Hz.

T1-weighted structural images were obtained using a Sigma Excite HD 1.5 *T* system (GE Yokogawa Medical Systems Ltd., Milwaukee, WI, United States). The generated MRI images comprised 166 sequential horizontal slices, each with a thickness of 1.2 mm, at a resolution of 512 × 512 points within a 261 × 261 mm field of view. The cortical surface was later reconstructed using the FreeSurfer Software (version 5.3, available at http://surfer.nmr.mgh.harvard.edu/).

### 2.5. Co-registration of MEG and MRI images

To facilitate a thorough representation of both the brain’s structural and functional activity, we performed a co-registration procedure to overlay MEG recordings onto individual MRI images; this is achieved by aligning the data based on specific marker locations.

We deployed five markers at the nasion, midline frontal region, vertex, and bilateral mastoid processes. Five corresponding coils were utilized for the MEG to generate a magnetic field. Conversely, we employed five lipid capsules for the MRI that appeared as high-intensity regions. The alignment of these markers enabled us to overlay MEG recordings onto MRI images with high precision, producing a cohesive picture of structural and functional brain activity.

### 2.6. MEG preprocessing

All data processing and analytical protocols were executed using Brainstorm (54) and FreeSurfer (55), supplemented by custom scripts developed in MATLAB R2021a (MathWorks, Natick, MA, United States). Preprocessing of the MEG data followed the procedures delineated in the Brainstorm tutorial, accessible at https://neuroimage.usc.edu/brainstorm/tutorials/.

Initially, the MEG data were down-sampled to 500 Hz, followed by a meticulous visual inspection to identify and exclude noisy sensors. The number of sensors removed was contingent on their condition during the examination, with up to 22 sensors discarded on a case-by-case basis. We applied notch filters (60, 120, and 180 Hz) to eradicate power-supply noise, followed by a band-pass filter (0.5–200 Hz). Independent component analysis was subsequently used to remove blinks and cardiac artifacts. Finally, any segments containing evident motion noise or radio frequency interference were visually identified by a MEG expert blinded to the participants’ identities and excluded from the analyses.

### 2.7. Source reconstruction and segmentation

We computed the head model using an overlapping sphere algorithm (56) with a lower-resolution cortical surface representation of 15,000 vertices. We used a weighted minimum norm estimation method to estimate the source orientation constraints (57). An identity matrix served as the noise covariance owing to the absence of noise recording. Signal sources were consolidated into 68 regions, represented in the Desikan–Killiany atlases (58), using principal component analysis to group the sources.

Following this, the data were segmented into continuous 5 s intervals. Each epoch was band-pass filtered for commonly utilized frequency bands: delta (2–4 Hz), theta (4–8 Hz), alpha (8–13 Hz), beta (13–30 Hz), and gamma (30–60 Hz). This preprocessing procedure aligned with our earlier studies (59, 60).

### 2.8. Mapping functional connectivity: the phase lag index

We used the PLI to estimate functional connectivity between signal sources. While functional interactions can be quantified by examining the phase relationship between their time series (61), it is crucial to recognize that reconstructed sources may contain artificial or spurious interactions owing to field spread. This field spread can result in the observation of artificial synchrony between proximate signal sources (62). This type of artificial synchrony can be mitigated by suppressing the zero-lag synchrony. As a zero-lag synchrony-insensitive interaction metric, the PLI effectively attenuated these artificial interactions (63).

In brief, the PLI was calculated for a given pair of signal epochs as follows. First, using the Hilbert transform, the instantaneous phase at each time point of the filtered waveform was determined for each signal. Then, the phase difference for the *k*-th timepoint, denoted as $\Delta \varphi \left(t_{k}\right)$ , was calculated for each time point. The PLI value between the two signal sources in an epoch was then obtained using the following definition (63):


$$PLI=\left|\frac{1}{N}\overset{N}{\underset{i=1}{\sum }}sign\left[\Delta \phi \left(t_{k}\right)\right]\right|$$

where *N* denotes the total number of time points in an epoch. We utilized PLI to estimate the phase synchrony between the source pairs for each frequency band. The PLI value ranges between zero and one, with a value closer to one indicating greater synchrony between the two sources.

### 2.9. Binary graph construction

A functional connectivity graph was constructed for each frequency band by calculating the PLI for each epoch and frequency range. This graph, which serves as a topological representation of a functional brain network, comprises nodes and edges. The nodes represent the brain regions, whereas the edges signify the functional connectivity between these regions, as quantified by the PLI values of the corresponding region pairs. Hence, each graph included 68 nodes corresponding to brain regions defined by the Desikan–Killiany atlas (58) and interconnected by weighted edges derived from PLI calculations.

An undirected weighted functional connectivity matrix (68 × 68) was constructed for each frequency band (delta, theta, alpha, beta, and gamma) and epoch. Next, we calculated the average matrices for each participant by combining all the epochs. We binarized the graphs using various thresholds to avoid including potentially spurious connections. Without a formal consensus on the optimal method for threshold selection, we followed previous studies (59, 60, 64) and set a proportional threshold κ, representing the proportion of total connections retained at 0.2; this implies that only the top 20% of connections were selected.

The most commonly used graph metrics for these binary matrices were calculated: clustering coefficient, characteristic path length, and SW (65). We obtained graph metrics for each frequency band (delta, theta, alpha, beta, and gamma) using different proportional thresholds. The Brain Connectivity Toolbox (http://www.brain-connectivity-toolbox.net/, ver. 2019-03-03) was used for this analysis. The mathematical definitions of these metrics have been reported previously (28).

### 2.10. Graph metrics: clustering coefficient, characteristic path length, and SW

Graph theory facilitates the comparison of different sets of graphs by translating complex global network properties into numerical measures, which can be statistically analyzed.

The clustering coefficient (C) is a measure that quantifies the extent to which nodes (or points) in a graph tend to “cluster” or form interconnected groups. Specifically, for a given node, it is the proportion of possible connections among the other nodes to which it is directly connected. A high C value in functional brain networks suggests a tendency for nodes to form tightly knit groups, indicating an organization of segregated neural processing (28).

Another key measure is the characteristic path length (L), the average shortest path length across all node pairs. The shortest path length between two nodes is the minimum number of edges required to travel from one node to another. Higher L values in functional brain networks suggest efficient information transfer within the network, indicating integrated neural processing (28). We calculated L only from the connected nodes to avoid an infinite path length when two nodes are disconnected, following the methods used in earlier studies (59, 60, 64).

SW is a key concept that combines the clustering coefficient and characteristic path length to evaluate overall network efficiency. A high clustering coefficient and a low characteristic path length characterize the SW, indicating an optimal balance between functional segregation and integration in the network. The SW is computed as the normalized clustering coefficient (C/Crand) ratio to the normalized characteristic path length (L/Lrand). To calculate the normalized C and L, we generated a set of 1,000 random graphs with the same number of nodes and edges as the original graph. In these random graphs, edges were randomly assigned via 1,000 rewiring iterations to maintain the same number of nodes and edges but eliminate any specific structure of the original graph. Crand and Lrand represent the average C and L, respectively, calculated over 1,000 random graphs (65); this ensured that any specific topology of the original graph did not bias the normalization process. We compared our measured network characteristics with those of an equivalently sized but randomly structured network.

### 2.11. Statistical analysis

Our study has three main objectives. First, we aimed to evaluate the impact of AD (relative to healthy controls) and different experimental conditions (EO vs. EC) on SW while accounting for potential age effects. Second, we sought to determine whether SW in the functional brain network was associated with delayed recall after adjusting for age. Finally, we investigated whether changes in SW across experimental conditions correlated with delayed recall, again controlling for age.

We commenced with linear mixed-effects analyses predicting SW based on disease condition (AD vs. control), experimental condition (EO vs.EC), and age. These were considered fixed effects, with subject-specific random intercepts incorporated to account for individual variability. The analysis was performed separately for each frequency band (delta, theta, alpha, beta, and gamma). We deliberately chose not to apply corrections for multiple comparisons across different frequency bands. These analyses were pre-planned, and SW across adjacent frequency bands, although quantifying different aspects of the brain network, were not expected to be strictly mutually independent (66, 67). For instance, SW derived from the alpha band network might exhibit similarities to SW obtained from networks in adjacent frequency bands (i.e., theta and beta). Therefore, we set the threshold for statistical significance at *p* < 0.05.

Subsequently, we conducted linear regression analyses to explore the association between SW in each frequency band and the delayed recall scores, as measured by the WMS-R delayed recall subtest. In these models, the delayed recall scores and age were incorporated as fixed effects to predict SW. Notably, the analysis was subdivided to individually assess the SW in each frequency band (delta, theta, alpha, beta, and gamma). Within each frequency band, separate analyses were performed for each disease group (AD and control) and each experimental condition (EO and EC). In this context, we opted not to apply corrections for multiple comparisons across the different frequency bands and experimental conditions. Our decision was based on two considerations. Firstly, there exists potential interdependency of SW across neighboring frequency bands, as mentioned earlier. Secondly, we recognize that SW data derived from the EO and EC conditions might not be strictly independent due to the expectation that SW from the EO condition would inherently resemble, to some extent, SW from the EC condition. This is because of the iterative nature of the measurements. Furthermore, both sets of data originate from the same subjects. Consequently, to maintain the rigor of our analysis, we restricted the application of multiple comparison corrections to the comparisons between the two disease groups. Accordingly, we established a threshold for statistical significance at *p* < 0.025 for these analyses.

Furthermore, we investigated whether changes in SW between the experimental conditions (EO vs. EC) correlated with delayed recall. We used a linear regression model to predict the relative difference in SW between the two conditions, calculated using the formula ([EC−EC]/[EO + EC]). Again, we used delayed recall scores and age as fixed effects to predict the relative differences. Following the previous strategy, we applied multiple comparison corrections for the two disease conditions, with statistical significance at *p* < 0.025.

To provide a comprehensive perspective, we conducted an exploratory analysis in which we expanded our analyses to include regression models predicting both the clustering coefficients (C) and characteristic path lengths (L) using the same approach that was initially applied to predict SW.

Lastly, we replicated the procedures using two distinct sets: one composed of patients with AD on donepezil medication and control subjects, and another comprising patients with AD not on donepezil medication and control subjects. It is important to note that this information primarily serves as a reference, considering the limited sample size of each group (seven subjects in the AD group without donepezil and twelve in the AD group with donepezil). Nevertheless, this approach enables readers to compare the effect sizes between patients with and without donepezil medication and the control subjects.

Before running the regression models, we validated the assumptions for the regression analysis using standard methods (e.g., testing for the normal distribution of residuals and homogeneity of variance). Visual inspection of the residual plots revealed no significant deviations from normality. However, the assumption of variance homogeneity was violated in some models. Hence, we use heteroscedasticity-robust standard errors in these models (68). All statistical analyses were performed using Stata software (Stata ver. 15.0; Stata Corp., College Station, TX, 750 United States).

## 3. Results

One participant from the AD group, who was undergoing donepezil medication, was excluded from the analysis because of excessive noise from unforeseen equipment malfunction. Consequently, our study included 42 participants: 24 healthy controls and 18 patients with AD. The participant characteristics are outlined in Table 1. Statistical comparisons revealed no significant differences between the two groups regarding age, sex, or years of education. Additional information regarding the characteristics of patients with AD, stratified into those receiving donepezil treatment and those not receiving it, is summarized in Supplementary Table S1. Similar analyses also found no significant differences between these two subgroups with respect to age, sex, years of education, or cognitive test scores.

**Table 1:** Demographics, clinical characteristics, and cognitive test scores of study participants.

	Control (*N* = 24)	AD (*N* = 18)	*T* or χ^2^	*p*
Demographics				
Sex (Male/Female)	15/9	11/7	0.01	0.927
Age	68.4 (6.9)	71.8 (6.2)	−1.67	0.104
Education years	12.1 (3.0)	11.6 (2.6)	0.59	0.559
Clinical characteristics (AD group only)				
Duration of illness	–	1.80 (1.1)	–	–
Cognitive test scores				
MMSE	28.8 (0.9)	22.0 (3.6)	8.93	<0.001
WMS-R attention and concentration index	105.0 (11.7)	87.8 (16.0)	4.03	0.002
WMS-R general memory index	100.7 (10.5)	66.1 (12.8)	9.62	<0.001
WMS-R verbal memory index	100.0 (11.0)	70.5 (11.6)	8.40	<0.001
WMS-R visual memory index	102.1 (11.4)	67.4 (14.3)	8.75	<0.001
WMS-R delayed recall index	98.8 (11.7)	60.8 (11.1)	10.66	<0.001

Numbers represent means (standard deviations) or counts. MMSE: Mini-Mental State Examination, WMS-R: Wechsler Memory Scale-Revised.

### 3.1. Effect of AD and experimental conditions on SW

**Figure 1:**
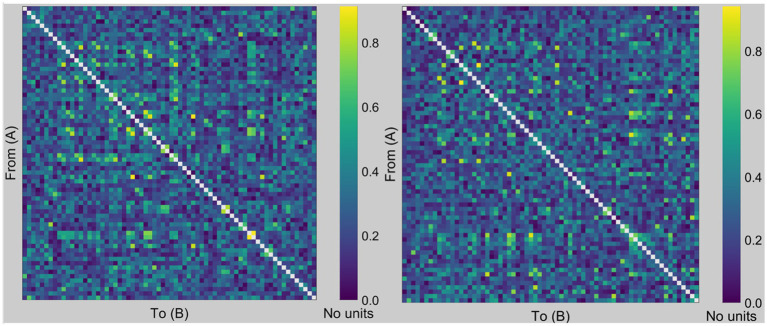
PLI imaging. Examples of PLI-based undirected weighted functional connectivity matrices. This figure illustrates representative graphs of the PLI from a chosen subject utilized for binary graph construction by applying a 20% threshold. We then computed the small-worldness, characteristic path length, and clustering coefficient from the derived binary graph, where the rows and columns corresponded to the respective nodes (i.e., regions of interest in the Desikan-Killiany atlas). For example, the value at the intersection of rows 10 and 15 signifies the PLI value between the right frontal pole (region 10) and the right temporal pole (region 15). The left figure was created using data from the eyes-open condition, whereas the right figure was created using data from the eyes-closed condition.

Figure 1 shows the PLI-based undirected weighted functional connectivity matrix (68 × 68) for a representative participant, finalized after applying a threshold of 20%. Subsequently, we compute the SW, characteristic path length, and clustering coefficient.

We developed a model predicting the SW for each frequency band, considering the disease condition (AD vs. control), experimental condition (EO vs. EC), age, and individual variation represented by random intercepts. Disease condition (*z* = −2.16, *p* = 0.031) and age (*z* = 2.21, *p* = 0.027) were significant predictors of SW in the gamma band. While the disease condition did not significantly predict SW in the other frequency bands, age was a significant predictor in the alpha (*z* = 2.07, *p* = 0.038) and beta (*z* = 2.52, *p* = 0.012) bands. These results are summarized in Table 2.

**Table 2:** Linear mixed-effects model coefficients predicting small-worldness in different frequency bands.

Frequency band	Coefficient	Robust Std. Error	*z*	*p*	Effect size
Delta					
Disease condition	−0.059	0.048	−1.22	0.221	−0.255
Experimental condition	−0.073	0.050	−1.45	0.148	−0.317
Age	−0.001	0.004	−0.15	0.879	<0.001
Theta					
Disease condition	−0.020	0.069	−0.29	0.771	−0.067
Experimental condition	0.078	0.061	1.27	0.205	0.259
Age	0.005	0.006	0.86	0.390	0.009
Alpha					
Disease condition	−0.056	0.078	−0.71	0.477	−0.193
Experimental condition	0.031	0.048	0.64	0.520	0.106
Age	0.011	0.005	2.07	0.038*	0.030
Beta					
Disease condition	−0.114	0.090	−1.27	0.203	−0.379
Experimental condition	−0.023	0.032	0.71	0.475	0.077
Age	0.014	0.006	2.52	0.012*	0.156
Gamma					
Disease condition	−0.132	0.061	−2.16	0.031*	−0.580
Experimental condition	0.018	0.032	0.55	0.580	0.078
Age	0.011	0.005	2.21	0.027*	0.030

This table displays each model predictor’s coefficients, robust standard errors, and *z*- and *p*-values. Statistical significance (*p* < 0.05) is indicated by asterisks (*). Effect sizes for dichotomous variables (i.e., experimental condition and disease condition) are calculated using a measure analogous to Cohen’s *d* (69), while effect sizes for continuous variables are determined by Cohen’s *f*^2^ (70). According to Cohen’s guidelines (71), values of *d* = 0.2 and *f*^2^ = 0.02 represent small effect sizes; *d* = 0.5 and *f*^2^ = 0.15 represent medium effect sizes; and *d* = 0.8 and *f*^2^ = 0.35 represent large effect sizes.

In our exploratory analysis, we sought to determine whether these effects on SW were influenced by the disease and experimental conditions affecting either C or L. To address this, we developed another model. This model predicted either C or L for each frequency band using the same approach as previously employed to predict SW. The experimental condition was a significant predictor of L in the delta band (*z* = 2.18, *p* = 0.029) and of C in the beta band (*z* = −2.37, *p* = 0.018). However, neither the disease condition nor age significantly predicted C or L in any other frequency bands. These results are detailed in Supplementary Table S2.

For reference, we conducted the same procedures with two distinct sets: one comprising patients with AD on donepezil medication and control subjects, and another including patients with AD not on donepezil medication and control subjects. Supplementary Table S3 presents these additional findings.

Interpreting these findings, we observed that individuals with AD demonstrated lower SW in the gamma band than the healthy controls. Moreover, older age was associated with higher SW in the alpha, beta, and gamma bands. However, the experimental conditions’ effect on SW was insignificant for any frequency band. Our exploratory analysis suggests that these effects cannot be attributed to the influence of disease and experimental conditions on either C or L.

### 3.2. Association between SW across various frequency bands and delayed recall in WMS-R, controlling for age

We conducted a linear regression analysis to investigate the relationship between SW across different frequency bands and delayed recall, as evaluated using the delayed recall subtest of the WMS-R. This analysis was performed separately for each disease condition (AD and control) and experimental state (EO and EC).

In the AD group, in the EO condition, the delayed recall scores of the WMS-R significantly predicted SW in the delta (*t* = −3.64, *p* = 0.002), alpha (*t* = −3.47, *p* = 0.003), and beta bands (*t* = −2.67, *p* = 0.018). Age significantly predicted SW in the alpha (*t* = 4.62, *p* < 0.001) and beta (*t* = 2.52, *p* = 0.023) bands. However, no significant predictors were identified under the EC condition.

Conversely, the control group did not show any significant predictors of an EO state. However, in the EC condition, delayed recall scores on the WMS-R significantly predicted SW in the alpha band (*t* = 3.38, *p* = 0.003). These findings are summarized in Table 3.

**Table 3:** Linear regression analysis to investigate the relationship between small-worldness across different frequency bands and delayed recall.

Group	Condition	Frequency		Coefficient	Robust Std. Error	*t*	*p*	η^2^
Alzheimer’s disease	Eyes-open	Delta	Delayed recall	−0.129	0.004	−3.64	0.002*	0.455
			Age	0.017	0.008	2.19	0.045	0.300
		Theta	Delayed recall	−0.009	0.007	−1.31	0.209	0.117
			Age	0.018	0.009	2.10	0.053	0.147
		Alpha	Delayed recall	−0.013	0.004	−3.27	0.003*	0.272
			Age	0.032	0.007	4.62	<0.001*	0.402
		Beta	Delayed recall	−0.011	0.004	−2.67	0.018*	0.173
			Age	0.026	0.010	2.52	0.023*	0.257
		Gamma	Delayed recall	−0.007	0.005	−1.48	0.160	0.119
			Age	0.014	0.006	2.28	0.038	0.155
	Eyes-closed	Delta	Delayed recall	−0.003	0.003	−1.02	0.322	0.030
			Age	−0.001	0.008	−0.17	0.865	0.002
		Theta	Delayed recall	0.004	0.004	0.88	0.392	0.019
			Age	−0.003	0.013	−0.23	0.824	0.004
		Alpha	Delayed recall	0.001	0.004	0.25	0.809	0.002
			Age	0.001	0.012	0.08	0.940	0.001
		Beta	Delayed recall	−0.005	0.004	−1.04	0.315	0.043
			Age	0.013	0.008	1.53	0.147	0.092
		Gamma	Delayed recall	0.000	0.006	0.01	0.994	<0.001
			Age	0.013	0.008	1.52	0.150	0.099
Controls	Eyes-open	Delta	Delayed recall	−0.002	0.003	−0.70	0.491	0.015
			Age	−0.001	0.007	−0.14	0.889	0.001
		Theta	Delayed recall	0.001	0.004	0.31	0.762	0.002
			Age	0.007	0.009	0.79	0.438	0.021
		Alpha	Delayed recall	0.002	0.006	0.25	0.808	0.003
			Age	0.010	0.008	1.22	0.236	0.042
		Beta	Delayed recall	0.009	0.005	1.89	0.072	0.098
			Age	0.017	0.008	2.18	0.041	0.109
		Gamma	Delayed recall	−0.001	0.003	−0.41	0.684	0.004
			Age	0.014	0.008	1.69	0.106	0.142
	Eyes-closed	Delta	Delayed recall	0.008	0.004	1.96	0.063	0.116
			Age	−0.004	0.007	−0.61	0.549	0.013
		Theta	Delayed recall	0.009	0.004	1.96	0.063	0.100
			Age	0.001	0.009	0.17	0.868	0.001
		Alpha	Delayed recall	0.012	0.004	3.38	0.003*	0.202
			Age	0.012	0.007	1.70	0.105	0.073
		Beta	Delayed recall	0.009	0.005	1.64	0.116	0.102
			Age	0.014	0.008	1.72	0.100	0.085
		Gamma	Delayed recall	0.003	0.004	0.60	0.556	0.015
			Age	0.007	0.007	1.00	0.329	0.040

This table displays each model predictor’s coefficients, robust standard errors, and *t*- and *p*-values. Statistical significance (*p* < 0.025) after applying Bonferroni correction is denoted by asterisks (*). The effect size is calculated using Eta-squared.

For our exploratory analysis, we aimed to determine whether the effects on SW were influenced by delayed recall and age on either C or L. To address this, we developed another model. This model, which predicted either C or L for each frequency band, followed the same approach as previously used for SW. In the EC state of the AD group, age significantly influenced C in the alpha band (*t* = 3.54, *p* = 0.003). However, neither delayed recall nor age exhibited significant predictive power for C or L in any other bands in the EO state. Furthermore, no significant predictors were identified for the control group in either the EO or EC state. Detailed results can be found in Supplementary Table S4.

For reference, we replicated the procedures for the AD group with two distinct sets: one focusing exclusively on patients with AD taking donepezil medication, and the other on patients with AD not taking donepezil medication. These findings are available in Supplementary Table S5.

In summary, our data revealed contrasting results. In the AD group, higher delayed recall scores correlated with lower SW across various frequency bands (i.e., delta, alpha, and beta) but only in the EO condition. In contrast, in the healthy control group, higher delayed recall scores correlated with higher SW but only in the alpha band and during the EC condition. These significant associations varied depending on the experimental conditions and showed diametrically opposing directions between the AD and healthy control groups. The exploratory analysis suggests that these observed effects cannot be attributed to the influence of delayed recall and age on either C or L.

### 3.3. Association between relative difference in SW across various frequency bands and delayed recall in WMS-R, controlling for age

We conducted a linear regression analysis to explore the relationship between the relative differences in SW (i.e., EO–EC)/(EO + EC) across different frequency bands (delta, theta, alpha, beta, and gamma) and delayed recall scores from the WMS-R. This analysis was performed separately for each disease condition (AD and control groups).

In the AD group, delayed recall scores from the WMS-R significantly predicted only the relative difference in SW in the alpha band (*t* = −2.98, *p* = 0.009). No other significant predictors were identified in this study.

In the control group, delayed recall scores from the WMS-R tended to correlate with the relative difference in SW in the delta (*t* = −2.35, *p* = 0.029) and alpha bands (*t* = −1.96, *p* = 0.063). However, after applying the Bonferroni correction, these correlations failed to reach statistical significance. The detailed results are presented in Table 4.

**Table 4:** Linear regression analysis to investigate the relationship between the relative difference in small-worldness across different frequency bands and delayed recall.

Group	Frequency		Coefficient	Robust Std. Error	t	p	η^2^
Alzheimer’s disease	Delta	Delayed recall	−0.005	0.003	−1.86	0.082	0.149
		Age	0.009	0.006	1.53	0.146	0.149
	Theta	Delayed recall	−0.006	0.003	−1.96	0.069	0.122
		Age	0.008	0.006	1.35	0.196	0.087
	Alpha	Delayed recall	−0.006	0.002	−2.98	0.009*	0.205
		Age	0.011	0.005	1.94	0.072	0.220
	Beta	Delayed recall	−0.002	0.001	−1.67	0.115	0.146
		Age	0.005	0.003	2.02	0.062	0.191
	Gamma	Delayed recall	−0.003	0.002	−1.71	0.109	0.143
		Age	0.000	0.003	0.15	0.887	0.001
							
Controls	Delta	Delayed recall	−0.004	0.002	−2.35	0.029	0.111
		Age	0.003	0.003	0.79	0.437	0.016
	Theta	Delayed recall	−0.003	0.002	−1.70	0.104	0.052
		Age	0.003	0.003	0.84	0.409	0.017
	Alpha	Delayed recall	−0.004	0.002	−1.96	0.063	0.188
		Age	−0.001	0.002	−0.56	0.584	0.005
	Beta	Delayed recall	0.001	0.001	0.42	0.681	0.006
		Age	0.001	0.002	0.31	0.762	0.003
	Gamma	Delayed recall	−0.001	0.001	−0.74	0.470	0.024
		Age	0.003	0.001	1.76	0.093	0.043

This table displays each model predictor’s coefficients, robust standard errors, and *t*- and *p*-values. Statistical significance (*p* < 0.025) after applying Bonferroni correction is denoted by asterisks (*). The effect size is calculated using Eta-squared.

In our exploratory analysis, conducted with the aim of determining whether the effects on the relative differences in SW were influenced by delayed recall and age, concerning either the relative differences in C or L, we constructed an additional model. This model, designed to predict the relative differences in C or L across each frequency band, employed the same methodology as previously used for the relative differences in SW. This study did not reveal other statistically significant predictors. Further details can be found in Supplementary Table S6.

For reference, we repeated the procedures within the AD group, differentiating between two subsets: one exclusively focusing on patients with AD receiving donepezil medication and another solely on patients with AD not receiving donepezil medication. These results can be reviewed in Supplementary Table S7.

In summary, our data highlight a notable correlation within the AD group. Delayed recall scores were found to significantly influence the relative differences in SW, particularly within the alpha band. Specifically, patients with AD patients with lower delayed recall scores exhibited increased reactivity in the SW of functional brain networks. Our exploratory analysis substantiates the notion that this observed effect cannot be attributed solely to the influence of delayed recall on the relative differences in either C or L.

## 4. Discussion

This study aimed to enhance the current understanding of functional brain networks in AD. Our first objective was to evaluate the impact of AD (in comparison to healthy controls) and different experimental conditions (EO vs. EC) on the SW of functional brain networks. The second aim was to explore the potential correlation between the SW in these networks and delayed recall. Finally, we intended to explore whether differences in SW under the two experimental conditions were correlated with delayed recall. After analyzing the PLI-based undirected binary graphs of 24 healthy controls and 18 individuals with AD, we discovered a pattern of diminished SW in the gamma band among individuals diagnosed with AD compared with healthy controls. We observed an association between advanced age and increased SW across the alpha, beta, and gamma bands. Nevertheless, the experimental conditions (EO vs. EC) did not significantly affect SW in any frequency band.

Our data revealed a divergent pattern regarding the relationship between delayed recall and SW in functional brain networks. Specifically, in the AD group, higher scores on the delayed recall component of the WMS-R correlated with diminished SW across multiple frequency bands (i.e., delta, alpha, and beta); however, this was only observable under the EO condition. In the AD group, advanced age significantly predicted increased SW in the alpha and beta bands; however, this was only observable under the EO condition. Conversely, enhanced delayed recall scores were associated with increased SW in the healthy control group, although this was only evident in the alpha band under EC conditions. These significant correlations fluctuated depending on the experimental conditions and exhibited inverse trends between the AD group and healthy controls.

**Figure 2:**
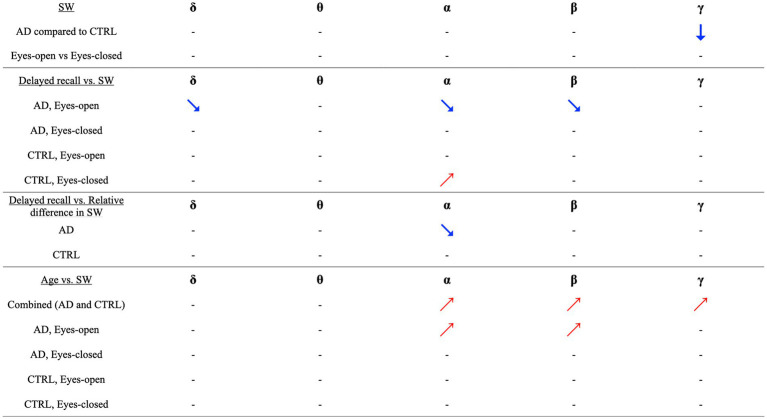
Visual and Correlational Insights into Small-Worldness (SW) and Delayed Recall in Alzheimer’s Disease and Healthy Controls. This figure succinctly illustrates key findings concerning the relationship between small-worldness (SW) in functional brain networks, Alzheimer’s Disease (AD), and delayed recall performance. Downward arrows symbolize the observed reduction in SW among AD patients compared to healthy controls (CTRL). Upward-right-pointing arrows suggest a positive correlation, while downward-right-pointing arrows indicate a negative correlation. Our primary observations are as follows: (i) Difference in SW between AD and CTRL: A significantly lower gamma-band SW was identified in individuals with AD, compared to CTRL. (ii) Impact of Experimental Conditions (Eyes Open [EO] vs. Eyes Closed [EC]): No significant effect of experimental conditions on SW was observed across any frequency band. (iii) Correlations between Delayed Recall and SW in AD Group (EO condition): In the AD group, Delayed Recall scores were negatively correlated with SW across delta, alpha, and beta bands. This effect was exclusive to the EO condition. (iv) Correlations between Delayed Recall and SW in CTRL Group (EC condition): In the CTRL group, Delayed Recall scores showed a positive correlation with SW in the alpha band, but only under the EC condition. (v) Delayed Recall and relative difference in SW (AD group): A notable correlation was observed where lower Delayed Recall scores in the AD group were linked with a more pronounced difference in SW between the experimental conditions. (vi) Correlation between age and SW (Combined group): A significant positive association was identified between age and increased SW across the alpha, beta, and gamma bands. (vii) Correlations between age and SW in AD Group (EO condition): In the AD group, higher age predicted an increase in SW within the alpha and beta bands, exclusively observed during the EO condition. These findings illuminate the intricate relationships and contrasts between SW, age, experimental conditions, and cognitive performance in AD and CTRL groups. It’s important to note that all correlations and directional relationships are context-specific and can vary across frequency bands and experimental conditions.

Finally, we investigated whether there was a correlation between the contrast in SW across the two experimental conditions and delayed recall. The results showed a significant correlation only within the AD group, in which delayed recall scores from the WMS-R significantly predicted the relative difference in SW in the alpha band. Specifically, patients with AD and lower delayed recall scores displayed more pronounced reactivity in functional brain networks. No other significant predictors were identified in this study. These results are summarized in Figure 2.

### 4.1. Gamma SW AD < healthy controls

Our study revealed a reduction in SW within the gamma band in individuals diagnosed with AD compared to healthy controls. Our exploratory analysis suggests that this effect cannot be solely attributed to the presence of AD on either C or L. This finding aligns with prior research conducted by Vecchio et al. (38) and Miraglia et al. (43), who similarly reported a decrease in SW among patients with mild cognitive impairment who eventually developed AD, compared to non-converters. This observation contradicts other graph-theoretical studies that reported no significant difference in SW between patients with AD and healthy controls (37, 40–42, 72, 73). Such contradictions may arise from different experimental conditions. Our study used the phase-lag index to measure connectivity, a departure from previous studies that employed diverse measures, such as lagged linear coherence (37, 41, 42, 72, 73) and synchronization likelihood (40).

Furthermore, our study and another previous study employed binary undirected graphs, whereas the remaining studies used weighted, undirected graphs (40). There was also a distinction in the normalization of the SW. Our study and another study (40) employed randomly generated graphs for normalization. In contrast, most studies used the average value over all frequency bands for normalization (37, 41, 42, 72, 73). A significant discrepancy lies in the definition of the gamma bands. Previous studies defined the gamma band as ranging from 30 to 45 Hz (37, 40–42, 72, 73), whereas we expanded its upper limit to 60 Hz. This modification could be a decisive factor in the discrepancy between our results and those of the previous studies. Although power spectrum analysis studies have highlighted an altered response near 30–45 Hz in patients with AD compared to healthy controls (e.g., higher response to specific tasks at 40–48 Hz (74) or 30–45 Hz (75) for AD), there is evidence suggesting the involvement of higher frequencies in memory function. For example, van Vugt et al. reported substantial increases in 48–90 Hz gamma power with working memory load (76), Griffiths et al. observed an increase in 40–50 Hz gamma power during episodic memory retrieval (77), and Carr et al. posited that 20–50 Hz gamma power and synchrony across the dorsal CA3 and CA1 networks are critical for coordinated memory reactivation across the hippocampal network (78). Given the significant memory function alterations in AD, our expanded definition of gamma may provide a more accurate representation of the atypical AD brain network underlying memory disturbances, which may remain undetectable with a narrower gamma definition. Based on these observations and considering that SW embodies an optimal balance between segregation and integration (34), the observed decrease in gamma band SW in AD could indicate an atypical structure of the memory-related brain network within higher-frequency bands (76–78). Concurrently, the marked discrepancies between our findings and previous research underscore the need for future studies to clarify these inconsistencies, perhaps by systematically comparing different connectivity measures and SW normalizations and examining the impact of expanding or narrowing the gamma band frequencies on the observed network characteristics.

### 4.2. Advancing age and increased SW, primarily in AD under EO

Interestingly, our study identified a correlation between advancing age and increased SW across alpha, beta, and gamma bands in the older population. However, a more in-depth analysis revealed that this trend was primarily apparent in the AD group under the open-eye condition. In these conditions, linear regression models indicated that advancing age significantly predicted increased SW in the alpha and beta bands. Our exploratory analysis suggests that these effects cannot be ascribed to the influence of age on either C or L. To the best of our knowledge, the only other investigation into the impacts of aging on resting-state brain networks was conducted by Hou et al. (45). They assessed 15 healthy young participants (average age: 23.1 years) and 10 healthy senior individuals (average age: 64.0 years). Their findings indicated that older groups exhibited significantly lower SW in the resting-state brain network. It is important to note that this effect was limited to the gamma band, and the participants’ eyes (open or closed) status during recording was not specified. It is challenging to directly compare our findings with those of Hou et al. owing to differing experimental methodologies (they utilized EEG, constructed a PLI-based weighted undirected graph, and defined the gamma band as 30–45 Hz, whereas we used MEG, constructed a PLI-based binary undirected graph, and extended the gamma band definition to 30–60 Hz). The results from our study and those of Hou et al. can be consolidated as follows: in a healthy population, younger adults demonstrate higher SW in their brain networks within the gamma band than their older counterparts. However, when examining only older demographics, the relationship between age and SW was insignificant in healthy individuals.

In contrast, in patients diagnosed with AD, the converse correlation is evident in the alpha and beta bands; advancing age is associated with increased SW. We suggest that the significantly lower SW in the older population than the younger population and the lack of a significant correlation between age and SW within the healthy older population may indicate a potential “floor effect” in this association. In contrast, observations from the AD group offered compelling insights. Patients with AD characteristically experience deterioration in non-memory-related abilities such as attention (79) and sensory function (80). Concurrently, alpha oscillations have been linked to attention control (81–84), and beta oscillations have been associated with sensory processing and attention (85, 86). Since SW is an indicative measure of a network’s efficiency (34), the inverse relationship between age and SW in AD suggests a compensatory mechanism. This mechanism could potentially counterbalance the age-associated decline in non-memory abilities, including attention and sensory functions, by improving the efficiency of the brain network. In contrast, healthy individuals with optimal non-memory functions may not require such compensatory network enhancements, thus underlining the unique network dynamics associated with AD.

### 4.3. Higher delayed recall and diminished SW in AD under EO in delta, alpha, and beta bands

Our study is the first to establish a correlation between diminished long-term memory performance, as indicated by lower delayed recall scores and increased SW across the delta, alpha, and beta frequency bands in patients with AD. However, this relationship was solely observed under the EO condition. Our exploratory analysis indicates that these effects cannot be solely attributed to the influence of delayed recall on either C or L. This observation aligns with the “two-stage” theory of memory, which suggests a dialogue between the hippocampus, where initial memory traces are formed, and the neocortex, where these traces are eventually stored for long-term retention (87, 88). Animal studies have shown that the coordination between these two brain structures may involve various oscillations crucial for memory consolidation, such as delta waves (0.1–4 Hz) (89, 90) and thalamocortical spindles (10–20 Hz) (90–93). These oscillations correspond to our study’s delta, alpha, and beta bands, in which we noted a significant correlation between long-term memory performance and SW. Studies on humans have emphasized the role of these oscillations in long-term memory formation. Jiang et al. demonstrated that successful long-term memory encoding corresponds to decreased alpha power in the sensory region of the attended target and increased power in the region of the ignored target, thereby suppressing distractions during the rehearsal period (94).

Similarly, Kaiser et al. identified significant positive correlations between frontal and parietal beta powers and delayed memory recall (95). Additionally, through time-frequency analysis, Axmacher et al. found that reduced slow hippocampal activity in the delta frequency range supported long-term memory formation (96). Our findings extend this literature by suggesting that not only do spectral powers within these frequency bands matter but that the SW of the network arising from these oscillations also plays a critical role in long-term memory performance, particularly among AD patients with AD. The inverse correlation between diminished long-term memory performance and increased SW across the delta, alpha, and beta bands in patients with AD could indicate a compensatory mechanism. Higher SW, indicative of a more efficient network structure (34), may partially act as a buffer against deficits in long-term memory performance by enhancing brain network efficiency. The lack of a significant correlation between delayed recall and SW in healthy controls and lower delayed recall in patients with AD suggests a possible “ceiling effect” for this association; this could imply that healthy long-term memory function may not require compensatory network efficiency, further highlighting the unique network dynamics at play in AD. Based on this hypothesis, future investigations could employ techniques designed to modulate the structure of functional brain networks to explore potential compensatory mechanisms. For example, non-invasive brain stimulation techniques such as transcranial direct current stimulation (97) and transcranial alternating current stimulation (98) could be useful tools. This approach provides a deeper understanding of the complex dynamics of network efficiency in the context of AD and memory performance.

### 4.4. Higher delayed recall and increased SW in healthy controls under EC, only in alpha band

The findings in the EC condition painted a notably different picture. We observed a significant correlation between improved delayed recall and increased SW; however, this was only observed in the alpha band and exclusively in the healthy control group. Our exploratory analysis suggests that these effects cannot be attributed to the influence of delayed recall on either C or L. Given the established role of alpha oscillations in delayed recall (90–93) and the premise that higher SW signifies a more efficient network structure, these results may not be entirely unexpected. However, the stark contrast between the EO and EC conditions might be more compelling. Our findings align with evidence from EEG and MEG studies, suggesting that whether the recordings are performed with eyes open or closed significantly influences the measures of functional connectivity and topological structure of resting-state brain networks (99–101); this underscores the crucial role of experimental conditions in interpreting and comparing the results of functional brain network studies. Given the observed differences in brain networks under EO and EC conditions, exploring the degree of variability or “reactivity” between these two states in the context of functional brain networks presents an enticing avenue for research. Guided by this interest, we proceeded to the final part of our study to investigate whether there was any correlation between the contrast in SW across the two experimental conditions and delayed recall performance.

### 4.5. Higher delayed recall and the relative difference (open-closed/open+closed) in SW in the alpha band

We explored the potential correlation between the variation in SW across the two experimental conditions (EO and EC) and delayed recall. While previous research has advocated the application of graph theory to EO EEG reactivity (99, 102–104), to our knowledge, our study is the first to probe the association between cognitive function and the reactivity of the functional brain network in patients with AD. Our findings presented an intriguing pattern: AD patients with lower delayed recall scores displayed more pronounced reactivity in the functional brain network of the alpha band. Importantly, this effect cannot be solely attributed to the influence of delayed recall on either the relative difference of C or L. This contradicts the findings of power spectrum analyses, which have typically associated AD with a progressive deficiency in the reactivity of alpha power (specifically, an insufficient reduction in alpha power upon eye-opening). Moreover, weaker reactivity has been linked to severe cognitive impairments (23). Therefore, one can deduce the importance of reactivity in alpha oscillations upon eye-opening; patients with AD exhibiting more severe symptoms would also show enhanced reactivity in the functional brain network and reduced reactivity in power spectral analysis, specifically within the alpha band. The progression of AD may thus involve changes in the brain’s alpha oscillation reactivity to the EO state, not merely by attenuating amplitude reduction (as suggested by power spectrum analysis) but also by amplifying the redistribution of phase-synchronized alpha oscillations across the entire brain (as evidenced by the SW of the functional brain network). In this context, our study not only corroborates but also broadens the existing evidence, suggesting that altered reactivity in alpha oscillations can be a prominent AD feature, observable in the power spectrum and the functional brain network within the alpha band. Both the reactivity in the power spectrum and the functional brain network epitomize distinct aspects of brain function, each offering invaluable insights into the characteristics of the AD brain. Our findings highlight the role of altered reactivity within the alpha band and underline the importance of evaluating both the power spectrum and functional brain network reactivity when studying AD, as both would offer distinct yet complementary insights.

### 4.6. The effects on SW cannot be simply ascribed to variations in C or L

An intriguing aspect of our study’s findings pertains to C and the L, both of which are foundational to the computation of SW. Despite our comprehensive analysis, we did not discern any significant influence of the predictors on either C or L that could directly account for the observed effects on SW. This raises a fundamental point: while SW inherently relies on both C and L, the variations we detected in SW do not simply mirror changes in these foundational metrics. This suggests that SW might capture specific network characteristics not exclusively reflected by C or L alone. This unique property of SW, as illuminated in our findings, emphasizes its potential as a sensitive and distinct measure of functional brain network organization, particularly in populations with neurodegenerative conditions such as AD. While SW is often considered in the context of its components, C and L, our study underscores the importance of regarding SW not merely as a derivative metric but as a standalone indicator with its own nuances and implications.

### 4.7. Limitations

While contributing valuable insights, this study has several methodological limitations. First, because of the relatively small sample size of 19 adults diagnosed with AD and 24 healthy controls, it is possible that the effects may have been overestimated (105). Future studies should consider larger sample sizes for a more precise estimation of the effect sizes of diseases and experimental conditions on the graph metrics. Second, we compared our results with those of other graph-theoretical studies that used different connectivity measures (e.g., PLI vs. lagged linear coherence), graph types (e.g., binary vs. weighted graphs), and definitions of frequency bands, which may not be entirely suitable; this also extends to studies that employed different numbers of regions of interest (nodes), as the number of nodes and edges influences graph metrics. It is important to note that this dependency becomes particularly significant when the node count falls below 200 (106). To date, no satisfactory methods have been proposed to address this factor. These limitations highlight the need for careful consideration of the design and interpretation of future studies considering the intricacies and nuances of graph-theoretical research.

## 5. Conclusion

In conclusion, our research aimed at expanding the current knowledge of functional brain networks in AD has successfully provided several key insights into the relationship between AD, experimental conditions (i.e., EC versus EO), SW in functional brain networks, and delayed recall. Our investigation revealed a decrease in SW in the gamma band among individuals diagnosed with AD compared with healthy controls, suggesting an unusual memory-related brain network structure within higher frequency bands. This observation, in contrast to previous studies, emphasizes the necessity for future research to address these discrepancies, potentially through systematic comparisons of different connectivity measures, SW normalizations, and evaluations of the effects of broadening the definition of gamma-band frequencies on network characteristics. Importantly, our study is the first to draw a connection between diminished long-term memory performance and increased SW across the delta, alpha, and beta bands in patients with AD, specifically under EO conditions. This observation supports the “two-stage” theory of memory and existing research on the role of these oscillations in memory formation but also extends the literature by suggesting the critical role of not just spectral power but also the SW of the network stemming from these oscillations for long-term memory performance. We propose that increased SW represents a compensatory mechanism that enhances network efficiency against memory deficits in AD. This insight opens new possibilities for future studies using non-invasive brain stimulation techniques to delve deeper into these potential compensatory mechanisms and their implications for network efficiency dynamics in AD and memory performance. Lastly, our study pioneered the exploration of the link between cognitive function and reactivity of the functional brain network in patients with AD. We found that AD patients with lower delayed recall scores showed stronger reactivity in the functional brain network within the alpha band under EO conditions, in contrast to earlier findings from power spectrum analyses. This finding suggests a potential change in the way AD progression influences the brain’s alpha oscillation reactivity to the EO state, not only by attenuating amplitude reduction, as suggested by power spectrum analysis but also by enhancing the redistribution of phase-synchronized alpha oscillations across the entire brain. Our work expands on existing evidence, highlighting that altered alpha oscillation reactivity, observed in both power spectrum and functional brain network analyses, is a significant AD characteristic; this emphasizes the importance of assessing both these aspects in studying AD, as they provide distinct yet complementary insights. Collectively, our findings contribute to a multifaceted understanding of functional brain networks in AD, emphasizing that the SW properties of these networks change according to disease status, cognitive function, and experimental conditions. We hope that our research serves as a platform for further studies in this area, enabling a deeper understanding of AD and driving the development of more effective management strategies.

## Data availability statement

The original contributions presented in the study are included in the article/Supplementary material, further inquiries can be directed to the corresponding author.

## Ethics statement

The studies involving humans were approved by the Ethics Committee of the Kanazawa University Hospital. The studies were conducted in accordance with the local legislation and institutional requirements. The participants provided their written informed consent to participate in this study.

## Author contributions

KY: Conceptualization, Data curation, Formal analysis, Investigation, Writing – original draft. TH: Formal analysis, Investigation, Methodology, Supervision, Writing – review & editing, Writing – original draft. DS: Data curation, Methodology, Software, Supervision, Writing – review & editing. NF: Data curation, Methodology, Writing – review & editing. MaK: Methodology, Supervision, Writing – review & editing. MS: Formal analysis, Investigation, Writing – review & editing. KK: Data curation, Writing – review & editing. MU: Data curation, Writing – review & editing. MiK: Conceptualization, Funding acquisition, Project administration, Resources, Supervision, Validation, Visualization, Writing – review & editing.

## Funding

The author(s) declare financial support was received for the research, authorship, and/or publication of this article. This study was supported by the Center of Innovation Program of the Japan Science and Technology Agency. The funder played no role in the study design, data collection, analysis, publication decision, or manuscript preparation. This research was partially supported by grants from the Moonshot Research and Development Program (grant number JPMJMS2297) of Japan Science and Technology.

## Conflict of interest

The authors declare that the research was conducted in the absence of any commercial or financial relationships that could be construed as a potential conflict of interest.

## Publisher’s note

All claims expressed in this article are solely those of the authors and do not necessarily represent those of their affiliated organizations, or those of the publisher, the editors and the reviewers. Any product that may be evaluated in this article, or claim that may be made by its manufacturer, is not guaranteed or endorsed by the publisher.
