# Supplementary material for: Eyes-state-dependent alterations of magnetoencephalographic connectivity associated with delayed recall in Alzheimer’s disease via graph theory approach
Source: Front Psychiatry. 2023 Oct 24;14:1272120. doi: 10.3389/fpsyt.2023.1272120 (PMC10628524; doi:10.3389/fpsyt.2023.1272120)
Supplement: Supplementary file 8 [file Table_8.DOCX]

**Supplementary Table 1: Demographics, clinical characteristics, and cognitive test scores of patients with Alzheimer’s disease**

Numbers represent means (standard deviations) or counts.
MMSE: Mini-Mental State Examination, WMS-R: Wechsler Memory Scale-Revised

**Supplementary Table 2: Linear mixed-effects model coefficients predicting characteristic path length and clustering coefficient in different frequency bands**

This table displays each model predictor's coefficients, robust standard errors, and z- and p-values. Statistical significance (P < 0.05) is indicated by asterisks (*). Effect sizes for dichotomous variables (i.e., experimental condition and disease condition) are calculated using a measure analogous to Cohen's d (1), while effect sizes for continuous variables are determined by Cohen’s f² (2). According to Cohen’s guidelines(3), values of d = 0.2 and f² = 0.02 represent small effect sizes; d = 0.5 and f² = 0.15 represent medium effect sizes; and d = 0.8 and f² = 0.35 represent large effect sizes.

**Supplementary Table 3: Linear mixed-effects model coefficients predicting graph metrics in different frequency bands, subgroup analysis**

This table displays each model predictor's coefficients, robust standard errors, and z- and p-values. Statistical significance (P < 0.05) is indicated by asterisks (*). Effect sizes for dichotomous variables (i.e., experimental condition and disease condition) are calculated using a measure analogous to Cohen's d (1), while effect sizes for continuous variables are determined by Cohen’s f² (2). According to Cohen’s guidelines(3), values of d = 0.2 and f² = 0.02 represent small effect sizes; d = 0.5 and f² = 0.15 represent medium effect sizes; and d = 0.8 and f² = 0.35 represent large effect sizes.

**Supplementary Table 4: Linear regression analysis to investigate the relationship between characteristic path length or clustering coefficient across different frequency bands and delayed recall**

This table displays each model predictor's coefficients, robust standard errors, and t- and p-values. Statistical significance (p < 0.025) after applying Bonferroni correction is denoted by asterisks (*). Effect sizes are calculated using Eta-squared.

**Supplementary Table 5: Linear regression analysis to investigate the relationship between graph metrics across different frequency bands and delayed recall, subgroup analysis**

This table displays each model predictor's coefficients, robust standard errors, and t- and p-values. Statistical significance (p < 0.025) after applying Bonferroni correction is denoted by asterisks (*). Effect sizes are calculated using Eta-squared.

**Supplementary Table 6: Linear regression analysis to investigate the relationship between the relative difference in** **characteristic path length or clustering coefficient** **across different frequency bands and delayed recall**

This table displays each model predictor's coefficients, robust standard errors, and t- and p-values. Statistical significance (p < 0.025) after applying Bonferroni correction is denoted by asterisks (*). Effect sizes are calculated using Eta-squared.

**Supplementary Table 7: Linear regression analysis to investigate the relationship between the relative difference in** **graph metrics** **across different frequency bands and delayed recall, subgroup analysis**

This table displays each model predictor's coefficients, robust standard errors, and t- and p-values. Statistical significance (p < 0.025) after applying Bonferroni correction is denoted by asterisks (*). Effect sizes are calculated using Eta-squared.

**References**

1. Westfall J, Kenny DA, Judd CM. Statistical power and optimal design in experiments in which samples of participants respond to samples of stimuli. J Exp Psychol Gen (2014) 143:2020–45.

2. Selya AS, Rose JS, Dierker LC, Hedeker D, Mermelstein RJ. A Practical Guide to Calculating Cohen’s f(2), a Measure of Local Effect Size, from PROC MIXED. Front Psychol (2012) 3:111. doi: 10.3389/fpsyg.2012.00111.

3. Cohen, J. (1988). Statistical power analysis for the behavioral sciences (2nd ed.). Hillsdale, NJ: Erlbaum.
